# On Cowpock in the Cow

**Published:** 1840-01

**Authors:** 


### Art. IV.

1. *Ueber Kuhpocken an Kühen.* Von E. Hering, Professor an der Königl. Thier-Arzneischule in Stuttgart, &c. *Mit einer colorirten Tafel.—Stuttgart,* 1839. 8vo, pp. 175.

*On Cowpock in the Cow.* By E. Hering, Professor at the Royal School of Veterinary Medicine, Stuttgard, &c. *With a coloured plate.—Stuttgard,* 1839.

2. *Praktische Abhandlung über die Wiedererzeugung der Schutzpockenlymphe durch Uebertragung derselben auf Rinder und andere impffähige Hausthiere.* Von Dr. Carl Gottlob Prinz, Professor der praktischen Thierheilkunde, &c. zu Dresden. *Mit zwei buntgedruckten Kupfertafeln.—Dresden,* 1839. 4to, pp. 42.

*A Practical Treatise on the Regeneration of the Protecting-pock (Vaccine) Lymph, by transferring it to Heifers and other domestic animals susceptible of inoculation.* By Dr. Charles Gottlob Prinz, Professor of Veterinary Medicine at the Royal Veterinary College, Dresden. *With two copperplate engravings.—Dresden,* 1839.

3. *Die Menschen- und Kuhpocken in ihrer Identität und Ruckbildung ersterer zur Vaccine, &c.* Von Dr. Basil Thiele, Inspektor der Medicinalbehörde in Kasan, &c. &c. (Henke's Zeitschrift, Erstes Vierteljahrheft, 1839.)—*Erlangen,* 1839. 8vo, pp. 26.

*On the Identity of Smallpox and Cowpox, and the Reproduction of the latter from the former, &c.* By Dr. Basil Thiele, Inspector of the Medical Board in Casan, &c. &c. (From Henke's Journal, No. I. 1839.)—*Erlangen,* 1839.

4. *Report of the Section appointed to enquire into the present State of Vaccination, read at the Anniversary Meeting of the Provincial Medical and Surgical Association, held at Liverpool, July* 25, 1839. —*Worcester,* 1839. pp. 98.

The state of security into which, in this country and in many of the continental states, medical practitioners as well as the public had been lulled with respect to smallpox, had led, as is but too commonly the case under like circumstances, to the entire neglect or careless employment of the very measures by which that security was induced. It is only through the repeated warnings of the enemy at the door, and the reappearance of the dreaded pestilence, even within the very lines of our presumed defences, that we are at length aroused to a sense of the dangers which threaten us, and to the study and investigation anew of those means of protection which have for a time availed us. The causes of the late renewed prevalence of smallpox are various; and, setting aside those cases in which no protective means whatever had been had recourse to, the number of instances of smallpox occurring, in which the operation of vaccination had been previously performed, is such as to demand a most anxious and searching investigation into the sources of failure Among those which have been assigned there are three especially worthy of consideration: 1, a presumed failure in the protection afforded by the vaccine after an interval of longer or shorter duration, and a consequent renewal of the previous susceptibility to smallpox infection; 2, a presumed deterioration in the powers of the vaccine lymph, arising, it is thought, from repeated transmission through the human subject; 3, negligence or carelessness in the performance of the operation, whether in the selection of the lymph used, or in ascertaining the healthy state of the recipient and the subsequent progress of the vesicle. The gradual failure of the protective powers of the vaccine, after a certain period of time, is an opinion pretty generally entertained in several parts of the continent, and we have recently had occasion to consider this point at some length, in connexion with its remedy, revaccination (vol. vii., p. 186). The question of a deterioration of the lymph in use has of late engaged much attention, as well in France and in this country as in several of the German states; and endeavours have been made in consequence to discover anew the vaccine disease in the cow, and to introduce fresh lymph, as it is supposed of a more active quality, from this source. The third source of failure, that of carelessness or the neglect of due precautions in the performance of the operation, is the explanation urged by those who are the more immediate followers of Jenner, and, especially, in the Report of the Vaccination Section of the Provincial Association, drawn up by its chairman, Dr. Baron. Whether one or all of these causes may not have been in operation is a subject for grave and unbiassed enquiry, and we hail with great satisfaction the exertions making both in this country and abroad to develope the truth.

From the Report of the Provincial Association it would seem that, in this country at least, the actual amount of failure has been much exaggerated; the cases of smallpox occurring after vaccination being scarcely, upon the whole, more in number than those occurring after a previous attack of smallpox, and the resulting mortality so small in these partially protected cases, as to be of little or no account in the general mass: but at the same time we must observe that there is an apparent bias in the report which gives to it too much of the air of special pleading; and although we are not disposed to question the general accuracy* of the conclusions arrived at by the author from the materials

* It is with regret that we must here refer to a serious inaccuracy on this very question, already pointed out by Dr. Gregory in the Medical Gazette for October last (p. 130). Our own personal experience accords with the statement made in the report of the not infrequent occurrence of secondary smallpox, though certainly not to the extent therein stated. We cannot but lament, therefore, that the want of accuracy in the reference to Mr. Crosse (*Report,* p. 64), has been the occasion of throwing some degree of discredit over other statements in the same document, which we believe to be substantially correct. If Dr. Baron, who is so thoroughly versed in this subject in placed in his hands, there is a want of precision in the summing up of the evidence, and of sufficient detail with respect to the individual questions circulated by the section, which render it, when considered as a statistical document, of less value than might have been expected. In other respects, however, information of the utmost interest and importance is brought before us, and more especially with reference to the actual nature of the vaccine, a subject worthy of every consideration, and to which we purpose to devote some attention in the course of the observations which we shall have to make upon the German works before us.

The treatise of Dr. Prinz is chiefly devoted to a consideration of the reproduction of the vaccine in the cow, and the means of effecting this object (retrovaccination), and of rendering the lymph thus generated applicable to the general purposes of vaccination. The work of Professor Hering, on the other hand, is intended to give a full account of the cowpock in the cow, and embraces the consideration of: I. The origin of the vaccine from other diseases. II. Retrovaccination. III. The spontaneous development of cowpock. IV. The influence of various external and internal conditions on the generation of the disease in the cow. V. The symptoms, process, &c. VI. The so-called spurious and anomalous cowpock. To these points of enquiry, taking the work of Professor Hering as our guide, we shall advert in succession.

I. *Origin of the Vaccine.* Four sources of the vaccine disease in the cow are mentioned by Prof. Hering: 1, the grease (Pferdemauke); 2, smallpox; 3, retrovaccination; and 4, spontaneous development. The last two of these we reserve for separate consideration, confining our attention at present to the presumed origin of the vaccine in the cow, first from the grease, or some other cutaneous disease in the horse, and, secondly, from the smallpox, as it occurs in man. The question, it should be observed, is perhaps the most important that can be entertained on the subject, as it involves the actual nature of the disease, its presumed identity with smallpox, and consequently the consideration of whether in its operation upon man, as a protective means, the action of the vaccine is that of an antagonizing power, more or less incompatible with the existence of variola, or a milder and safer modification of the same power, by which the tendency to the reception of variola is exhausted, in the same manner as by the introduction of the more severe and exquisite form of the disease.

It is unnecessary to enter into any lengthened discussion upon the origin of the cowpock from the grease, and more especially, since much confusion would seem to exist among continental writers as to the precise meaning of the term. Whether Professor Hering is correct in referring it to the disease to which he alludes under the name of *Pferdemauke*, we must leave to the veterinary surgeon to determine. It is now acknowledged that Jenner was mistaken in the name of the equine disease, to which he referred the origin of cowpock, though not in the all its branches, should have been led into this serious lapse of memory, it need not perhaps be a matter of surprise that other members of the section may have likewise been deceived; but we believe that the majority of its members never saw the report until it was in print, their duties being to obtain and transmit information to the secretaries, for the use of the chairman.

circumstance that the horse is subject to a vesicular eruption about the heels (but also found in other parts of the limbs and body), identical in its nature with the vaccine, and capable of generating the genuine vaccine vesicle by inoculation upon man or upon the cow. "It is ascertained," says Dr. Baron, "that the horse is liable to a vesicular disease of a variolous nature as well as the cow; and that lymph taken from the horse and inserted into man will produce an affection in all respects like that derived from the cow, and equally protective. The error consisted in believing that this affection was *the grease*, and that it required to be transmitted through the cow to give it efficacy. A misapprehension of this kind may well be excused in the infancy of so complicated an investigation; the disease appearing for the most part on the thin skin of the heels of the horse, and the traditions among the farriers in the country, leading to the mistake. We now know that the vesicle may appear on other parts of the animal's body; and that the horse as well as the cow has, in different ages and in different countries, suffered both from the mild and malignant *variolæ*." (*Vaccine Report*, p. 16.)

The *Pferdemauke* is described as consisting in an erysipelas of the skin of the ball and pastern joint, which not unfrequently extends also to the back of the metatarsus or metacarpus, and at first forms small vesicles, by the bursting of which an acrid lymph of peculiar smell escapes. These vesicles, through neglect, want of cleanliness, and improper treatment, readily pass into tetter-like fissures (the chronic form of the disease), difficult to heal, and finally giving rise to various degenerations of the skin and subjacent cellular tissue. (*Hering*, p. 5.) The disease, however, is elsewhere described as affecting the thighs and other parts. Several instances of the inoculation of cows with the matter derived from the vesicles of this equine disease, in its primary stage, are given, in which the vaccine eruption was generated.

The origin of the cowpock from smallpox is a question of far greater moment, and deserves the closest and most sifting investigation. "According to theory," says Professor Hering, "the identity of these two diseases is much more probable than that of the equine disease (*Mauke*) and the smallpox; but," he adds, "experience has not established the expectation." The opinion of the identity of the vaccine with variola, as is well known, was entertained by Jenner, who gave to the former affection the name of variolæ vaccinæ, and has since been strongly advocated by Wedekind, Dr. Baron, and others. This opinion, however, until lately, rested upon very insufficient grounds; and numerous unsuccessful attempts having been made to inoculate the cow and other animals with smallpox by Sacco, Ring, Dalton, and others, it might be considered, as far as negative evidence could decide, as entitled to but little weight. Dr. Baron it is true, in his Life of Jenner, devotes a considerable portion of the first volume to an attempt at establishing the identity of smallpox and the vaccine; but we think that a careful examination of his arguments, and of the historical details upon which they are founded, do no more than show that cattle have at various times been attacked with an eruptive disease, possessing all the characteristics of variola, the question of the identity of this affection with the vaccine resting still upon the grounds of analogy and presumption, rather than upon experimental proof, actual observation, or historical evidence. Dr. Sonderland, of Bremen, was, as far as we know, the first to point out a method of inducing the vaccine in the cow by the direct application of the smallpox virus. The object was effected by means of a woollen bed-cover, removed from the bed of a patient who had died in the suppurative stage of smallpox, and wrapped round the body of the animal. The account of Dr. Sonderland's experiment will be found in Hufeland's Journal for January, 1831 ; but the fact became disputed in consequence of repeated attempts subsequently made elsewhere to generate the vaccine in this manner having invariably failed. At Stockholm and Utrecht, however, a pustular eruption was observed to follow the application of the infected woollen cover on those parts of the body of the animal in immediate contact with it. This eruption, as observed at the former of these places, quickly dried up; at the latter, according to Numan, who watched the progress of the experiment, the vesicles appeared on the sixth day, and by the tenth had reached their full development, and contained a tolerably clear lymph, and then became incrusted with a brownish scab, which fell off in the course of two days. The affection was almost purely local, being attended with scarcely any constitutional disturbance. Four children were inoculated with the lymph with some appearance of success, but the result was not cowpock.

The results of attempts made to inoculate the cow with variolous matter, though contradictory, have proved, in some instances at least, more satisfactory. Dr. Gassner is said to have inoculated several cows with smallpox lymph, and in eleven of these cowpock was produced, from which four children were inoculated, with the effect of producing, not any modification of smallpox, but the genuine vaccine. But the most important of the results hitherto obtained respecting this question, are those elicited by the investigations of Dr. Basil Thiele, of Kasan, in South Russia, and Mr. Ceeley, of Aylesbury. Those of Dr. Thiele, although obtained some years since, were first published in January, 1839, by Dr. Henke, of Erlangen, in his excellent journal.

It appears that Dr. Thiele, after some fruitless attempts to induce the variolation of the cow, according to Dr. Sonderland's method, the failure of which is attributed to the experiments having been performed in the winter season, and without due regard to keeping up the temperature of the stall, in the spring of the year 1836 caused a cow to be inoculated with smallpox matter. The operation was performed by the assistant-physician, Fomin, and succeeded; several children were inoculated with the matter thus generated in the cow, and the lymph was transmitted to Dr. Thiele, in whose presence other children were inoculated. The progress and external characters of the eruption which ensued were altogether similar to the genuine vaccine, though accompanied with more severe constitutional symptoms. The matter thus obtained, at the time of Dr. Thiele's communication, had passed through seventy-five successive inoculations (*Impfgenerationen*), and had been employed in upwards of 3000 individuals. Dr. Thiele repeated the experiment, more immediately under his own personal inspection, in the spring of the year 1838, with similar success. The following directions are given as to the methods of procedure necessary to be followed, and are the more valuable, since by neglect of one or other of the precautions recommended, numerous experimental researches of the same description previously entered into were rendered abortive. 1. The cow should be a recent milker, between four and six years old, and should have a white udder, to admit of the pock being easily and clearly seen. 2. It must be kept in a warm stable (at about 15° Reaumur, = 66° F.), fed with its customary food, and regularly milked. 3. The hair must be shaved off, and the posterior part of the udder chosen for the insertion of the matter, that the animal may not be able to lick the spot. The incisions are to be made as in inoculating with cowpock, but deeper, and the part is to be bound up. 4. The lymph used may be taken either immediately from the pustule or from points charged not longer than from ten to twenty days previously; the pustules from which it has been procured must be clear, transparent, and of a pearly aspect, and the lymph itself limpid. "The greater or less malignity of the epidemic," says Dr. Thiele, "and of the individual case from which the lymph is taken, has no essential influence upon the vaccine generated; for in a case in which the smallpox eruption was confluent, became black, and the child died, a perfectly genuine vaccine was generated by the transmission." (p. 17.) With respect to the general and local symptoms in the inoculated cow, it is stated that, on the third day, a hardness was remarked in the cellular tissue of the udder; and on the fifth, a pustule resembling the vaccine was formed, which from the seventh to the ninth day contained a clear lymph, and had a central depression or pit; from the ninth to the eleventh day it began to dry up, a crust being formed, which, on falling off, left a small smooth cicatrix. From three to six incisions usually gave rise to only one or two pocks. Acceleration of the pulse and increased heat were noticed between the fourth and seventh days, but the general health and appetite were but little disturbed. The pocks generated in children from this lymph presented an appearance perfectly similar to the genuine vaccine, differing only in being somewhat more intense in the first inoculations.

These researches of Dr. Thiele do not however satisfy Professor Hering, who considers the identity of smallpox and the vaccine as not yet proved. He appears to attach far too much weight to the negative evidence derived from the repeated failures, although all such evidence ought to yield to one well-authenticated and unexceptionable fact. It is with much satisfaction that we are enabled to refer to the Report of the Vaccination Section for the confirmation and further extension of these researches by those of Mr. Ceeley, of Aylesbury.

"On the first of February, 1839, he (Mr. Ceeley) inoculated with smallpox matter (*variola discreta*), of the seventh or eighth day, three young heifers; a fourth was at the same time vaccinated. We shall not mention all the particulars connected with these examples, but select the first as an illustration of the admirable skill and judgment with which the process was conducted. Mr. Ceeley made seven punctures and introduced fourteen points near the left *labium pudendi*, and on the same day inserted two setons with matter from the same subject. On the ninth day after this process, he vaccinated the same animal on the right *labium pudendi*, with fifth, sixth, and seventh days' lymph from a child, in seven punctures with fourteen points; and below the pudendum in four punctures with eight points. On the tenth day after the insertion of the variolous matter, one of the punctures near the posterior margin of the left *labium pudendi* had assumed the form of the natural vaccine vesicle. By gently removing the central irregular crust, and carefully puncturing the cuticle, he was able, in the course of an hour, to charge thirty-eight points with lymph, and on the same and subsequent days to use part of it on children and adults. On the thirteenth day the smallpox vesicle was more inflamed and florid; this was the fifth day after the insertion of vaccine lymph, at which time all the eleven punctures were converted into effectual vesicles; from these he took fine clear lymph, and used it on children and adults. Both the variolous and vaccine vesicles subsequently ran nearly a parallel course; so that on the twenty-sixth day of the former, and the seventeenth day of the latter, the scars of both appeared perfectly similar. To obviate objections which might arise from the insertion of the vaccine lymph on the ninth day after the inoculation with the variolous matter, Mr. Ceeley reinoculated a sturk on the 15th of February with smallpox matter, of the seventh or eighth day, on the *labium pudendi*. He made eight punctures, which were deluged with the variolous fluid from capillary tubes. On the fifth day the four upper punctures were enlarged and elevated, the other four were less so. On the sixth day all presented the appearance of the vaccine vesicle. From one of them he took lymph with difficulty, and scantily charged thirty-nine points. On the eighth day he again took lymph from the vesicle opened on the sixth. On the ninth day the vesicles were enlarging, and he again opened carefully the first vesicle and charged twenty points. On the tenth day the four lower vesicles were increasing, and from them he charged twenty-seven points. After this time the brown crusts appeared, and the disease gradually declined. The animal was subsequently inoculated both with variolous and vaccine matter, but no result followed." (*Vac. Report*, pp. 26-8.)

Mr. Ceeley's experiments were conducted in the presence of five medical men and one veterinary surgeon; and he has twice succeeded in accomplishing his object after many previous fruitless trials. Portions of the lymph were transmitted to Dr. Baron, and the vesicles produced from it in every respect corresponded with those delineated by Jenner. "The correctness of the vesicle formed by it," says the report, "exhibits a marked contrast to that which we have seen produced by other virus now in use; and we fear that the local as well as general disturbance occasioned by the latter, so far from being a source of protection, will be found to be the reverse." (p. 26.) We have only to observe that these experiments, as far as we are able to judge of them from the accounts before us, appear to be highly satisfactory and conclusive; and we look upon the investigations of Dr. Thiele and Mr. Ceeley as the most important in their bearings upon the general subject of the vaccine which have been developed since the original researches of Jenner himself.

II. *Retrovaccination.*—The production of cowpock in the cow by inoculating with vaccine lymph taken from the human subject, has been had recourse to with different views at different periods since the first introduction of the vaccine. The essay of Dr. Prinz, as we have before remarked, is devoted exclusively to the consideration of this subject, and is divided into two parts:—the first comprising a general investigation respecting the regeneration of the cowpock lymph, the advantages to be derived by vaccination from the practice, with an historical account of its introduction; the second pointing out the means of generating the vaccine disease in the cow by inoculation from man, and the measures necessary to be adopted in the removal, preservation, and application of the lymph thus generated for the purposes of further vaccination.

The presumed advantages to be derived from the reproduction of the cowpock, by transference of the vaccine from man to the cow, are stated to be :—1, That it affords a means of proving the genuineness and efficiency of the lymph employed; 2, of increasing the supply of vaccine matter; 3, of keeping up the supply for future use; and 4, a method whereby the cowpock lymph may be renewed, whenever the same may become necessary. The uncertainty of success which has hitherto attended attempts at retrovaccination, together with the consequent expenditure of time and labour, are impediments to the practice upon any but the last of these grounds. With respect to the first, it may be stated that we have more practicable and equally sure modes of testing the efficiency of the vaccine lymph, where it is thought necessary, by revaccination, or by inoculation with smallpox matter; and, although the third of these presumed advantages has found an advocate in Numan, who assures us that he had succeeded in preserving the cowpock lymph, by inoculation from cow to cow, for sixteen weeks, we are informed by others that the success of the practice cannot be depended on beyond the fourth transplantation, and that, consequently, as a means of increasing or keeping up the supply of the vaccine, it must prove nugatory. There remains therefore for consideration only the supposed restoration of the vaccine lymph to its original purity and efficiency by the retransference of the lymph to the cow, and the consequent retransmission of the disease through the animal from which it was, in this form at least, originally derived. Dr. Prinz contends for the practice of retrovaccination upon this ground alone, and considers that the lymph thus attains renewed efficiency as a preservative against smallpox. He seems, with many other authorities at the present day, to be impressed with the opinion of a gradual deterioration of the lymph in use, and, although he does not consider it to have yet entirely lost its efficiency, he suggests the possibility that the powers of the lymph may become still further impaired, the vesicle produced gradually diminishing in size after repeated vaccinations, and the protection afforded to the constitution becoming less. This deterioration and loss of power in the lymph would seem, under certain circumstances, actually to occur; thus Meyer, among others, perceived that the vesicles produced from his original stock of lymph became, from year to year, smaller, affording an impoverished and less copious supply of lymph, the vaccination from which was not always successful; but having at length succeeded in procuring fresh matter from the cow, the resulting vesicles were large and turgid with lymph, and left a cicatrix perfectly resembling that of the original cowpock inoculations, while the vaccination succeeded in all cases, or at least with very rare exceptions. (*Prinz*, p. 9.)

The advantages of retrovaccination, however, are not to be estimated by those resulting from a recurrence to the cow for original or primary cowpock lymph, and, as it appears to us, Dr. Prinz and others of our continental brethren are reasoning and drawing inferences upon erroneous grounds, when they place the primary vaccine, no matter how generated, and the retrovaccine upon the same footing. Granting that the vaccine lymph in its repeated transmission through the human body has lost power, or acquired certain deteriorations impairing its original efficiency, it by no means follows that by the transference of this deteriorated or impoverished lymph to the cow, it should thereby become purified and increased in power, so as to resemble primary lymph. If also, as the experiments of Dr. Basil Thiele, confirmed and extended as they are by those of Mr. Ceeley, tend to show, the vaccine is in reality nothing more than a mild form of variola, and the lymph derived from the primary vaccine pustule in the cow generates a form of variolous disease in man, which protects, not by a presumed antagonism, but by its identity of action upon the constitution, a renewed transmission of the virus through the cow should, if it have any effect at all, tend to render the vaccine still milder in its operation, while the impurities presumed to be derived from the repeated transmissions of the lymph through the human subject, would probably only produce further modifications in the disease as it affects the cow, and become complicated with others derived from constitutional or acquired peculiarities in that animal. In order to establish any claim to our notice for this operation of retrovaccination, it must be shown, first, that the repeated transmission of the vaccine through the human body is actually and necessarily attended with a modification of its properties and a corresponding loss of its efficiency; and, secondly, that the retransmission of the humanized or deteriorated lymph through the cow is capable of removing the acquired impurities and renovating the weakened powers. The first of these propositions there may be some grounds to entertain ; but we would remark that hitherto neither of them, and least of all the last, has been made the subject of proof. The possibility of the vaccine becoming milder by repeated inoculation may be inferred from an analogous amelioration which is known to occur, as Professor Hering observes, in two other nearly allied forms of eruptive disease, smallpox and chickenpock. It would also seem to acquire some quality in its transmission by which it becomes assimilated to the human constitution, since the difficulty of inducing successful vaccination with primary cowpock lymph is much greater than with lymph which has already been passed a few times through the human body, though the constitutional and local effects of the primary lymph, where it succeeds, would seem to be more marked and severe. That a further reduction of its power or alteration of its nature takes place, where the necessary precautions have been used, does not however appear so decided: for although the cases of failure reported are, from whatever cause, numerous, still it must be borne in mind, that the lymph now in use in this country has passed, not through three or four, not through hundreds of individuals, but through thousands, and tens of thousands, while yet we hear of skilful and careful vaccinators expressing themselves, like Mr. Dodd, of Chichester, who states "that he has *not seen a single instance of smallpox in a patient whom he had vaccinated, though he had resided at Chichester ten years.*" (*Vaccination Report*, p. 48.) That the vaccine however may, through carelessness or want of skill, become deteriorated, does not admit of question. The salutary cautions, so earnestly enforced by Jenner, of attending to the state of the skin of those submitted to vaccination, more especially with respect to herpetic eruptions, bear closely on this point. The modifying powers of itch, whether in man or in other animals, are equally insisted upon by Professor Hering, yet from the work of Dr. Heim, noticed in our Seventh Volume, there seems to be a laxity of opinion in regard to this point which, if carried into practice, may possibly go far to explain many of the failures among continental vaccinators.

The question, however, still remains as to the efficacy of retrovaccination in correcting these evils. As it appears to us, the practice, until further experience has shown to the contrary, must be held of very doubtful utility; while under any circumstances the variolation of the cow, performed according to the methods so skilfully devised and clearly pointed out by Dr. Thiele and Mr. Ceeley, would seem to afford a preferable mode of obtaining lymph of renewed activity and power. Professor Hering thus expresses himself respecting retrovaccination:

"Opinions differ as to the value of the vaccine thus renewed; some at once admit the cowpock generated by means of retrovaccination to be of the same value as that primarily developed in the cow; others think that there is no difference in efficiency between this renovated vaccine and that hitherto in use, while Ritter even considers the inoculation of the cow from vaccinated children as a doubtful proceeding, since, according to his experience up to this time, lymph so obtained does not equal in activity that of spontaneous origin, and at the same time we are exposed to the danger of obtaining modified pocks in the cow, and in this manner disturbing the purity of the original source. If the vaccine during its transmission through so many individuals, who may be variously affected with the germs of cachexy, scrofula, or other diseases, shall really have lost in intensity or protective power, it remains to be proved whether, by a single transmission through a cow, it shall be again so far purified and strengthened as to be capable of being placed on a par with primary cowpock. Where, however, this last (primary cowpock) is not met with, and the vaccine hitherto in use shows a falling off of its essential properties, I hold a renovation of the same by means of retrovaccination to be advisable, and by no means participate in the fear of thereby giving rise to spurious pocks." (p. 23.)

Retrovaccination has been practised, as we are informed by Dr. Prinz, in his historical account, first, with the purely scientific aim of establishing the fact of the reproduction of the vaccine in the cow, as by Woodville, Coleman, Valentin, Frank, and other of the earlier cultivators and advocates of cowpock inoculation; secondly, with the view of keeping up and increasing the supply of lymph, as by Junker, Sacco, Magliari, and others; and, lastly, in later times, by many of the German inoculators especially, in the hope of renovating the powers of the vaccine. Very full directions are given by Dr. Prinz for the performance of the operation, and the precautions necessary to ensure success are carefully pointed out. These relate to the selection of the animal, its age, general state of health, condition of skin, &c., to the characters of the lymph employed, to the time of year, &c. Yearling cow-calves, young heifers, and young cows in calf three or four years old, with well-developed udders, the skin of which should be, if possible, without hair and colourless, are recommended as the fittest subjects for the operation. (*Prinz*, p. 31.) The lymph should be carefully selected from well-developed vaccine vesicles, and transferred immediately from the arm of a healthy child to the animal, and the most fitting season is from the commencement of the month of March to the end of June. The best mode of performing the operation seems to be by making longitudinal incisions, either upon the udder or on the teats, from a third to half an inch in length, the skin being previously placed upon the stretch with the left hand of the operator, and of such a depth that the edges of the wound may gape a little; the lymph is then carefully applied over the whole surface of the wound, and the animal properly secured. It is not necessary to enter into any account respecting the subsequent removal of the lymph, which may be generated in the pustules, and its preservation for use, for which full directions are given by Dr. Prinz, as these will readily suggest themselves to every experienced vaccinator, and by none other can the operation be attempted.

III. *Spontaneous Development of Cowpock.* Professor Hering remarks, in relation to the presumed connexion of the genuine cowpock with the *Pferdemauke* or grease, or rather with the peculiar eruptive disease in the horse, which was at one time mistaken for that affection, that this connexion is, on the continent, of very rare occurrence, and that almost all the instances of cowpock observed in the cow have arisen independently of any such affection. After the discovery of Jenner was promulgated, it was found that the vaccine disease not only showed itself in many places on the continent, but that also its occurrence among cows had been before noticed, and even that its protective powers against smallpox were known, although no practical inference had ever been drawn from the fact. In the Göttingen *Allgemeinen Unterhaltungen* for the 24th of May, 1769, it is said that the disease was not infrequent around Göttingen, that it attacked the milkers, and that such persons were considered to be protected against the smallpox. (*Hering,* p. 24.) Professor Hering quotes from the chronicles of Bishop Marius, of Lausanne, who lived in the sixth century, a passage which he thinks refers to cowpock: "*Anno* 570. *Hoc anno morbus validus cum profluvio ventris et variola Italiam, Galliamque valde afflixit. Et animalia bubula per ea loca maxime interierunt.*" However this may be, not long after the original discovery of the cowpock in Gloucestershire by Jenner, the vaccine disease was also observed in many of the southern and midland counties of England; in Ireland it is known to have occurred in the county of Cork, while at various times it has been noticed in most of the continental states of Europe. In the year 1800, Sacco saw the disease in the north of Italy, in cows which had been brought from Switzerland into the plains of Lombardy. In the year 1812 primary cowpock occurred in many places in northern Germany, and was observed by Dr. Bremer at Berlin, and by Dr. Fischer in Lunenburg. The disease has also been noticed, both before and subsequently to this last date, in Sleswich and Holstein, in Brunswick, Wirtemberg, Holland, Switzerland, Piedmont, Rome, and lastly in France. In May, 1812, Dr. Bremer, as we have stated, observed the disease among the cows in the neighbourhood of Berlin, and found the teats covered with brown horny crusts; he could discover clear lymph in two only of the vesicles submitted to his inspection. Two girls also had become infected with the disease. Dr. Bremer vaccinated twelve children with complete success from one cow and from one of the girls, and continued the inoculations with the regenerated lymph. Dr. Fischer, of Lunenburg, describes the cowpock which he observed in the cow in April and May of the same year as follows:

"The eruption showed itself on the teats of milking cows as small, blueish, or more correctly blackish-brown, glistening pustules, which, even from the commencement, were hard to the touch and covered with a thick skin, but about the eighth or ninth day became incrusted with a scab gradually increasing in thickness and hardness. These hard vesicles, which felt like small hazel nuts deeply seated in the skin, increased so much in from four to six days, that the largest of them, if isolated, attained the size of a *Sechsers* or a

*Groschen* (about that of a sixpence) in circumference. Their figure was round or oval; their contents at the commencement clear and resembling lymph, becoming more consistent at a later period. The cows suffered more or less severely (?); they were very sensitive during milking, but showed no constitutional disturbance." (*Hering*, p. 26.)

In Sleswich and Holstein, scarcely a year passes without the occurrence of cowpock among the cows. According to the report of Professor Ritter it becomes epidemic among the large herds of cattle, in a manner similar to the smallpox in man. It was however with difficulty, notwithstanding frequent opportunities of seeing the disease in the cow, that he could observe it in its perfect form, sometimes from receiving the information of the outbreak of the eruption too late, sometimes from the pustules having been ruptured in milking and degenerating into ulcers, or from their having been subjected to local treatment, by which the character of the eruption was destroyed. He thus often saw large herds of cows affected with the disease, without being able to find one from which he could obtain clear lymph. Inoculations with primary lymph were, however, successfully accomplished in children, in the years 1824, 26, 29, 30, and 32, which were accompanied by local effects of more severity than usual, but without any danger or injurious consequences. In March, 1836, a woman at Passy, in the vicinity of Paris, became infected from a cow with an eruption on the hands and face, which was recognized by M. Bousquet as the vaccine. In the same year genuine cowpock in the cow is said to have been observed also at Amiens and Rambouillet. The investigations carried on by the Académie Royale de Médecine, in connexion with the occurrence of the cowpock at Passy, and the circumstances attending the recent discovery and introduction of primary vaccine lymph, by M. Estlin, are already sufficiently known to our readers.

The instances of cowpock in the cow which have come under the notice of Professor Hering, in Wirtemberg, either personally, or through the medium of the official reports, are very numerous, and are classified by him as follows: 1. Instances of genuine cowpock in the cow, with successful transference of the same to man by inoculation. 2. Instances of probably genuine cowpock, the inoculation from which either failed or was not attempted. 3. Cases of accidental infection of man from primary cowpock. Of the first class, it appears that within the ten years from 1827 to 1837, there were sixty-nine instances of the occurrence of genuine cow-pock, in which eighty-four head of cattle are reported as being affected. From these 126 children, besides one young female, were inoculated with success, in thirty-six of whom, however, the further inoculation failed. In addition to these there were twenty-two successful and two unsuccessful inoculations, in which the precise number of children operated upon is not given. Of the second class, in which the inoculation either failed, or was for various reasons omitted to be performed, there were 152 instances, the number of cows attacked amounting to 208. In 107 of these the inoculation was attempted but failed; in forty-five the reporters were either unable to obtain lymph, on account of the pustules having proceeded too far, or abstained from the inoculation on account of the characters of the eruption varying, more or less, from the descriptions given by Jenner and Sacco. The instances of the third class reported were twelve in number, in addition to three others included among those belonging to the first class. In the able summary which the author gives of these cases, as well as in the more general notice of the instances of cowpock in other countries, which he refers to as being of spontaneous development, he has frequently pointed out the entire want of connexion with the *Pferdemauke*, or any other disease in the horse. We must, however, observe, that he has strangely overlooked the more important question, of ascertaining whether there may not have been traces of a connexion between small-pox in man and the occurrence of these instances of cowpock in the cattle of the respective localities in which it has been observed to occur.

IV. *Influence of various External and Internal Conditions on the Production of the Disease in the Cow.* From a careful collating of the cases of original cowpock belonging to the first and second classes of the special reports, Prof. Hering deduces some valuable results.

1. The influence exercised by the geognostical characters of the soil and elevation of surface, as far as these conditions extend within the limits to which the reports refer, appears to be null. The geological formations chiefly occurring, we may observe, are the Muschelkalk, Lias, and Jura limestones, and the Bunter and Keuper sandstones, both belonging to our new red sandstone series. With respect to elevation, it has been thought that from the prevalence of cowpock in Holstein and Sleswich, and other situations of like character, that it is a disease of the lower lands, an opinion to which the greater number of cases observed in the Neckarkreis, an extent of country, for the most part lying lower than the other three departments of the Danube, the Jagst and the Schwarzwald, (see *British and Foreign Medical Review*, Vol. VII., p. 187,) lends some countenance; but upon closer examination of the district reports, this conclusion is not borne out, since it is not the lower districts of the Neckarkreis, but those the situation of which is considerably elevated which afford the greater number of cases of cowpock in the cow. The author concludes, therefore, "that the more or less frequent occurrence of primary cowpock does not depend upon the geological formation, and that the disease is not more prevalent in the lower-situated and warmer districts; at the same time it is also not infrequent upon the mountains and in the most uncultivated (*rauhesten*) parts of the country." (p. 106.)

2. The author next considers the effect of in-door or out-door foddering, the latter of which is most usual in the north of Germany, and in our own country, even throughout the winter. The case would seem to be different in Wirtemberg, where, in consequence of the severity of the winters, the cattle are only kept out of the stall in summer and autumn; while, in many thickly-populated districts, the practice of out-door feeding has been long discontinued altogether. Many reasons, which it is unnecessary for us here to consider, have contributed to this practice of stall-feeding, and in the vineyard countries especially (the valley of the Neckar and several lateral valleys,) the cows only leave the stall for the purpose of being led to drink. It is in these places, says Professor Hering, that the greatest number of cowpock cases have been observed, while, in others, in which a greater number of cows are kept, which are out in the more favorable seasons of the year, hitherto only a few cases have been known. The stall fodder consists usually in summer of grass and clover, but among the cattle which are fed with the refuse of the breweries and distilleries, the eruption also occurs, as Professor Hering has himself observed; and he remarks that the cows of the great milk establishments in London, among which Woodville and Pearson had observed the disease, are fed especially with grains from the great breweries. A change of fodder from dry to green has already been assigned by Jenner as a cause of eruptions on the udder. A dry, and, usually towards the end of winter, sparing supply of fodder, observes the author, naturally tends to diminish the secretion of milk; if now in the spring a greater quantity of better and more succulent food is quickly obtained, there is a determination of fluids to the udder, and this it is chiefly which gives rise to the eruption. This peculiar state of the udder is further brought about by the practices of fraudulent dealers, who abstain from milking cows brought into the market with the express purpose of inducing distention of the udder, while the change of fodder, consequent upon the transfer of the animal to another owner, assists, as the author thinks, in the production of the eruptive disease. (p. 108.) Jenner, however, was of opinion that the eruptions thus generated were not genuine cowpock; for, according to his observations, the milkers who became infected from pustules so induced, were still susceptible of the attack of smallpox. With regard to the presumed effects of stall-foddering in the Neckarkreis, the author remarks that in that department, in consequence of the number of small proprietors who themselves attend to the cows, and to whom the government premium offered for the detection of primary cowpock is an object, the instances of eruptive disease in the cattle are readily brought forward, whereas in the Donaukreis, where the cattle owners are possessed of a greater number of cows, and intrust their herds to the care of children and labourers, the disease is not only less likely to come under their notice, but the reward offered for its detection is a matter of too little moment to induce them to make known its existence.

3. The following table exhibits the number of instances of genuine, and probably-genuine cowpock in the cow, reported from the year 1825 to 1837, inclusive:

| Year. | Cases of Genuine Cowpock. | Probably-Genuine Cases. |
|---|---|---|
| 1825 | 0 | 1 |
| 1827 | 2 | 3 |
| 1828 | 1 | 2 |
| 1829 | 14 | 24 |
| 1830 | 5 | 26 |
| 1831 | 7 | 24 |
| 1832 | 6 | 12 |
| 1833 | 5 | 9 |
| 1834 | 6 | 12 |
| 1835 | 7 | 12 |
| 1836 | 8 | 17 |
| 1837 | 8 | 10 |

Professor Hering attributes the apparent prevalence of cowpock, in the years 1829-30 and 31, to the circumstance of the public mind having been, in the first of these years, then only fully awakened to its existence, and instructed in its nature; and to the effect of the government premium in bringing these cases into notice. Subsequent to this period it will be observed, that the annual number of cases reported of each class has fluctuated but little, though considerably less in amount than those of the years specified. Whether, in the years alluded to, there was any epidemic influence in action appears doubtful, as it is not improbable that, after the first impression among the proprietors of the cattle had subsided, many instances of the eruption might occur without being brought before the notice of the district reporters.

4. The effect of season is shown by the subjoined table, which proves that the spring and commencement of summer, as Jenner had before remarked, are the most favorable to the development of the disease.

| Months. | Genuine Cases. | Cases presumed Genuine. |
|---|---|---|
| January | 2 | 5 |
| February | 3 | 5 |
| March | 5 | 11 |
| April | 6 | 21 |
| May | 18 | 23 |
| June | 13 | 18 |
| July | 6 | 9 |
| August | 5 | 13 |
| September | 2 | 20 |
| October | 2 | 10 |
| November | 2 | 7 |
| December | 5 | 10 |

The average monthly number is 5·75 of the first class of cases, and 12·66 of the second, or, taking both together, 18·4; consequently, the months of April, May, June, and September are those which are in excess of this average, while January and February are greatly below it. Were a similar result to obtain over a sufficiently extended number of instances, it might be inferred that temperature exerted a very decided influence upon the prevalence of the disease, the cold of the winter and the heat of the middle of summer being both opposed to its spread.

5. The effect of age upon the frequency of cowpock is very difficult to estimate with certainty; and from various causes it is not always possible to ascertain with precision the facts necessary to establish it. Hitherto it has been thought that cows calving for the first time were the most readily affected by the disease, and consequently also the best fitted for experiments upon inoculation. The results of such of the Wirtemberg reports as give the age of the cows, which include about one third of the number of instances observed, would seem rather to militate against this opinion. Of 108 cows affected, 3 were two years old; 26 between two and a half and three years (*primiparæ*); 13, four years; 17, five years; 24, six years; 13, seven years; and 12, from eight to ten years: but it is probable that some included within the later periods were more advanced in age than is stated in the reports. The period of milking would seem to be that during which the cows are most subject to cowpock on the udder; and it is obvious that the same cause which has been before alluded to as predisposing to attacks of eruptive disease, namely, a determination of blood to the udder, must operate here. Woodville and Ritter both assert that cows not giving milk are not liable to cowpock, and the Wirtemberg reports show that the eruption appeared, in a majority of instances, from four to six weeks to three months from the time of calving, but that instances were not infrequent in which old milkers, that is, from four to nine months from the time of calving, were also attacked. Exceptions however are mentioned to the general rule of milking cows only being liable; a cow in calf for the first time was attacked with genuine cowpock, probably however by infection from an older cow which had previously had the disease; another case occurred in a cow not milking, in which the disease is thought to have been spontaneously developed, and another in a two-year old, from which children were vaccinated with success. Upon the average, about two thirds of the affected cows, that is, those in which the time of milking is specified, were in the first period of milking, or from the time of calving to the third month inclusive; the remaining third were old milkers.

It will be observed that, in preparing the foregoing abstract, we have restricted ourselves to the matter of our text. It is, however, obvious, that if we admit even the *possibility* of the derivation of the disease in the cow from human smallpox, (and we presume that much more than this must be now admitted,) the admission not merely greatly diminishes the value of the statistical details given, but introduces a new element into the proposition which alters the whole aspect of the case. All future enquirers into the statistics of cowpox in the cow must make the contemporaneous prevalence or non-prevalence of smallpox in man a principal subject of attention.

v. *Symptoms. Progress of the Disease, &c.* Genuine cowpock in the cow is thought by some authors to be attended by constitutional symptoms of some severity, so that it has been attempted to draw a diagnosis between the genuine and spurious eruptions by the presence or absence of febrile and other constitutional symptoms. The distinction is not however found to hold good, since spurious eruptions are frequently found to be attended with constitutional symptoms, and the genuine cowpock, as Jenner himself observed, may be attended with little or no disturbance. The opinion however would seem to prevail so strongly in Wirtemberg that, in many cases of primary cowpock, the physicians state that, from the want of fever and of general symptoms, or from some irregularity in point of time in the outbreak of the eruption, they have been induced to consider the disease as spurious, and consequently to refrain from experimental trials of inoculation therewith. At the same time it is to be remarked, that in several cases of inoculation from cowpock eruptions, not agreeing in these respects with Sacco's description, successful results have been obtained. In many of the reports nothing positive is given upon this point, but in seventeen cases of the first class, (namely, that in which the genuine nature of the disease was confirmed by subsequent inoculation, and the production of correct vaccine vesicles,) it is stated that the fever was wanting, while it is expressly stated to have been present in twenty-four cases only, and is commonly described as slight. Loss of appetite was remarked in thirty-six cases. Among the instances belonging to the second class (that in which the attempts at inoculation failed), there were only fifteen in which fever was observed to be established; in nine cases it was doubtful, and in thirty is said to have been altogether wanting. Rumination without food (*Wiederkauen bei leerem Maul*) was observed in only six of the cases; dimness of the eyes, restlessness, and other symptoms mentioned by Sacco, were as rarely, or even more rarely noticed. The most frequent constitutional symptom mentioned is a diminished and sometimes vitiated secretion of milk; but even genuine cases are reported in which the secretion of milk had been in nowise disturbed. Little dependence, however, as Professor Hering remarks, is to be placed upon the reports in this respect, since in many cases the information given could only be derived from the cow-proprietors, or their servants, the accuracy of whose testimony the temptation of the offered premium tends much to bring into question. The author concludes therefore that, since fever, loss of appetite, diminished secretion of milk, &c., are not always present, but, on the contrary, are often altogether wanting in the genuine cowpock, the existence of constitutional disturbance can no longer be received as a criterion of the genuine character of the eruption.

To come now to the local symptoms: the eruption, according to the Wirtemberg reports, was found, in by far the greater number of cases, to be seated on the teats, but in several instances on the udder also; in a few it was confined to the udder alone. This differs somewhat from the opinion of Jenner, who mentions only the teats as the seat of the genuine pustules, and still more from the assertion of Ritter, "that the pustules never break out on the udder, but always on the teats only, the papulæ which appear on the udder forming no cellular pustules, but only vesicles of a whitish or yellowish colour, which empty themselves entirely on being punctured." The number of the pustules was commonly of little importance, with the exception of a few instances in which from twenty to thirty pocks are mentioned as having occurred. The severity of the local inflammation, and the consequent sensibility of the part, were pretty generally found to vary with the greater or smaller number of the pustules. The size of the pocks is most frequently described as equalling that of a pea; sometimes it is compared to that of a lentil, or vetch. In a few cases, the pustules are said to have been as large as a horse-bean, a kreuzer, pfenning, or groschen, (that is, about equal in circumference to a sixpence,) and in one case as large as a hazel-nut. The form of the pustules was most frequently observed to be round and level-topped, with a slight depression or pitting in the centre. Deviations from this however occurred, the central depression being often scarcely to be seen, and frequently wholly wanting. In the place of this depression, hitherto considered as characteristic, in a few cases a mere dusky point was observed; but in others the pustule was raised in the centre and consequently conical, remained flat, or, by a change taking place in its progress from the flattened form, at length became pointed. The cellular structure of the pustule is always mentioned as occurring in the genuine affection, and would seem to be the most constant criterion by which to distinguish this from the spurious eruptions, which are usually simply vesicular. From this character Sacco drew the inference, that the genuine cowpock was seated in the subcutaneous cellular tissue, whereas the spurious pocks being easily lacerated by the touch, and discharging their contents at once, were, he presumed, to be merely in the epidermis. "The structure of a pock," says Professor Hering, "may be best compared to the pulp of a lemon, the cells of which must be lacerated or cut before they will discharge their contents. Hence it also happens that, although the primary cowpocks appear to contain but little lymph, when they are opened, or the crust is removed, more flows out than had been expected." (p. 122.) Some variation appears to have existed with respect to the colour of the pustules, without there being any reason on this account to suspect their genuine character. This, we are aware, is contrary to the opinions of some high authorities, and we are willing so far to concede to these opinions as to say, that, until this and some other points of difference in the characters of the more recent cowpock eruptions from those assigned to the Jennerian pustule have been more closely investigated, lymph should by no means be taken from such pustules for the purposes of vaccination. It is even possible that some of the recent failures in the protecting powers of vaccination may have arisen from a want of due attention to the scrupulously correct characters of the pustules or vesicles from which lymph has been taken. The Wirtemberg reports describe the colour of the cowpock pustules as being in some cases blueish-white, or blueish; in others silvery, or with a lustre resembling mother-of-pearl, and lead-coloured; instances are however not unfrequently mentioned in which the pustules were white, whitish-yellow, pale-yellow, or of the colour of pus; and the author remarks that pustules which at the first glance appeared to be white or yellow, became silvery or pearly, on putting the finely wrinkled skin on the stretch. (p. 123.) The remarks which we have made with respect to the colour of the eruption will equally apply to the state of the areola, which, in several of the Wirtemberg cases, was observed to be very pale, or was altogether absent. Inoculation was however performed from these cases, and it is said with successful results. The author observes as follows, in reference to this point:

"In place of the erythematous inflammation in the most superficial layers of the skin is frequently found a somewhat deeper-seated inflammation of the cutis, and probably even of the subjacent cellular tissue and neighbouring glands. In this case, little or no redness is perceived, but, on the contrary, a hard and painful tumefaction (*Wulst*) is felt surrounding the pustule, and even a distinct hardened knot in the substance of the udder. There is here most probably a circumscribed inflammation of the udder, but which, if many such pustules be thronged together, may be sufficiently important to give rise to symptoms of fever, to affect the secretion of milk, and to render the milking of the animal almost impossible." (pp. 123-4.)

It is a matter of some difficulty to determine with sufficient accuracy the progress of cowpock, since in most cases the animal is seen only once, and the reports of the proprietors and herdsmen are of course not to be depended upon. In cases of retrovaccination and of inoculation of the cow with primary lymph, the first signs of the success of the inoculation became apparent on the sixth day; in the accidental infection of healthy cows through milking, the first trace of the pock is said to have appeared on the third or fourth day, and is compared to the mark of a fleabite. Pustules thus generated attained their full development on the eighth, ninth, or tenth day, then became turbid and formed a crust, which remained to the third or fourth week, leaving a whitish cicatrix on falling off. The contents of the genuine pock are a clear, inodorous lymph, the consistence of which is sometimes perfectly limpid, sometimes a little clammy. The lymph, though at one time thought to retain its transparency uninjured, is said to become turbid and puriform after the full development of the pustule. It would seem, however, to be essential to the success of vaccination that only the clear colourless lymph should be employed. In the Wirtemberg reports only two exceptions are mentioned to this rule, in one of which the lymph was bloody, and in the other is described as being opaque and curdled (*klümprich*). It should be remarked that when the pocks are ruptured and irritated from repeated milking, they are longer in healing, but, according to the reports, do not give rise to malignant or fretting ulcers, as mentioned by Jenner. The formation of the scab commences in the central portion of the pustule, and, as the edges of the pock gradually contract, the crust is of course smaller in circumference than was the pustule the place of which it occupies. It is usually pretty thick, has a darkish-brown colour, the external layer being the darkest; it is thick, horny, and transparent at the edges, and the inner surface is of a pale or yellowish-brown colour. The cicatrix, as far as it has been examined, presents nothing characteristic.

Before quitting this subject, it is necessary to refer to the irregularity observed in the appearance of the pocks. The simultaneous development of the whole eruption has hitherto been held to be an essential characteristic of genuine cowpock; but here, as in some other particulars, the Wirtemberg reports are at variance with received opinions. In very many instances, it is said, that individual pustules were as much as from eight to fourteen days later in their appearance than the general eruption, and that, notwithstanding, these pustules were as available for the purposes of inoculation as those which first appeared. Among the sixty-nine instances belonging to the first class, in no less than twenty-three, or one third of the number, was the eruption of the pustules not simultaneous. In one case nearly four weeks elapsed from the first outbreak of the eruption, during which fresh vesicles from time to time made their appearance. We need scarcely point out that the same grounds for caution in the employment of lymph from sources of this description exist, as from those in which other hitherto presumed irregularities take place.

It is unnecessary to dwell upon the unusual symptoms which were remarked in a very few instances, and only as occurring in rare exceptional cases. Among these, however, it may be stated that the rumination with the mouth empty, sometimes observed before the coming out of the pock, is attributed by the author to the presence of a vesicular eruption within the mouth. Among the irregularities is also mentioned an instance of genuine cowpock occurring in a cow, which, according to its owner, had already, some years before, suffered several times from a similar eruption. Eleven months later, the same cow was again attacked with an eruption, the nature of which, however, through failure of the experimental inoculation, could not with certainty be established; we may mention that the characters of this second eruption, as given in the detailed report, are to us by no means satisfactory.

VI. *Spurious and Anomalous Cowpock.* In the preceding abstract, which we have condensed from the treatise of Professor Hering, it has been our main object to lay before our readers the substance of the Wirtemberg reports, so far as they relate to the symptoms and progress of cowpock in the cow. The author places in a fourth section those cases reported upon, which, on account of their deviation from the characters assigned to the genuine disease, cannot be classed with any variety of cowpock, and must be considered as either spurious and anomalous in their nature, or essentially different affections. Among these he successfully describes: 1, The *Spitspocken*, *Nachpocken*, or *Euterseuche* (miliary-pock, after-pock, and udder eruption). These he considers to be varieties of the same affection, and differing from genuine cowpock, not only in the characters of the eruption, but in the far greater rapidity of their progress, by which at all times they may be readily distinguished. These eruptions, especially the two former varieties, which are named by Professor Hering respectively, *variolæ vaccinæ miliares* and *variolæ vaccinæ secundariæ*, are sometimes found to occur in connexion with the genuine cowpock, and we may observe that the *v. v. secundariæ* would seem to bear the same relation to the true vaccine in the cow which the miliary eruption, sometimes observed to precede and accompany the development of the vaccine vesicle in children, bears to the vaccine in man. 2, The *Stein-pocken*, or *Warzen-pocken* (stonepock or wartpock), *v. v. tuberculosæ*, and *v. v. verrucosæ* of Viborg. There are also varieties of the same affection frequently occurring together, and essentially distinguished from genuine cowpock by the form and structure of the eruption. The pocks are dry, hard, and remain for weeks or months partly in the form of warts, with brownish, rugged, cracked tops, partly as firm fatty tubercles. 3, The *Wasser-pocken*, or *Wind-pocken* (waterpock, or bladderpock), *variolæ vaccinæ albæ* of Jenner. These are simple elevations of this cuticle, and are readily characterized by their purely vesicular nature, as essentially different from the cellular pustule of genuine cowpock. Other eruptions noticed are the *v. v. herpeticæ* of Viborg, and the amber, blue, and black pocks of Nissen. These last have been already referred to in our Seventh Volume, and need not detain us here; as to those who attentively study the characters of the true vaccine, the marked deviations exhibited by these eruptions from the correct cellular pustule will always afford a sufficient warning to avoid them as sources of lymph.

The close analysis we have given of the work of Professor Hering is sufficient in itself to mark our estimate of its merits, and we cordially recommend it to the careful study of all who are desirous of contributing to the elucidation of the many important questions connected with the vaccine, as containing a vast store of valuable materials for reference and study.

